# Supplementary material for: Hospital-at-home care in Singapore: A review of overseas protocols and guidelines to support implementation and policy redesign (systematic review)
Source: PLoS One. 2025 Jun 11;20(6):e0325662. doi: 10.1371/journal.pone.0325662 (PMC12157091; doi:10.1371/journal.pone.0325662)
Supplement: S1 Table — (DOCX) [file pone.0325662.s001.docx]

**APPENDIX S1**

| Search Syntax for Database Search and Advanced Internet Search | | |
| --- | --- | --- |
| Database | No | Search |
| PubMed | #1 | "recommendation*"[Title/Abstract] OR "polic*"[Title/Abstract] OR "implement*"[Title/Abstract] OR "requirement*"[Title/Abstract] OR "protocol*"[Title/Abstract] |
|  | #2 | "Home Care Services"[MeSH Terms] OR "Telemedicine"[MeSH Terms] OR "Hospital in the home"[Title/Abstract] OR "Hospital at home"[Title/Abstract] OR "home hospital"[Title/Abstract] OR "early supported discharge"[Title/Abstract] OR "home based care"[Title/Abstract] OR "remote care"[Title/Abstract] OR "virtual ward"[Title/Abstract] OR "virtual hospital"[Title/Abstract] OR "virtual care"[Title/Abstract] OR "bed substitution"[Title/Abstract] OR "Telehealth"[Title/Abstract] OR "hospital based home care"[Title/Abstract] OR "home hospitalisation"[Title/Abstract] |
|  | #3 | "Subacute Care"[MeSH Terms] OR "Acute Disease"[MeSH Terms] OR "Inpatients"[MeSH Terms] OR "Internal Medicine"[MeSH Terms] OR "Family Practice"[MeSH Terms] OR "Geriatrics"[MeSH Terms] |
|  | #4 | Combined Search:  ("Home Care Services"[MeSH Terms] OR "Telemedicine"[MeSH Terms] OR "Hospital in the home"[Title/Abstract] OR "Hospital at home"[Title/Abstract] OR "home hospital"[Title/Abstract] OR "early supported discharge"[Title/Abstract] OR "home based care"[Title/Abstract] OR "remote care"[Title/Abstract] OR "virtual ward"[Title/Abstract] OR "virtual hospital"[Title/Abstract] OR "virtual care"[Title/Abstract] OR "bed substitution"[Title/Abstract] OR "Telehealth"[Title/Abstract] OR "hospital based home care"[Title/Abstract] OR "home hospitalisation"[Title/Abstract]) AND ("Subacute Care"[MeSH Terms] OR "Acute Disease"[MeSH Terms] OR "Inpatients"[MeSH Terms] OR "Internal Medicine"[MeSH Terms] OR "Family Practice"[MeSH Terms] OR "Geriatrics"[MeSH Terms]) AND ("recommendation*"[Title/Abstract] OR "polic*"[Title/Abstract] OR "implement*"[Title/Abstract] OR "requirement*"[Title/Abstract] OR "protocol*"[Title/Abstract]) Filters: English, from 2014 - 2023  475 Results |
| Scopus | #1 | (TITLE-ABS-KEY ( "geriatric" ) OR TITLE-ABS-KEY ( "family practice" ) OR TITLE-ABS-KEY ( "internal medicine" ) OR TITLE-ABS-KEY ( "inpatient" ) OR TITLE-ABS-KEY ( "acute disease" ) OR TITLE-ABS-KEY ( "subacute care" ) ) |
|  | #2 | (TITLE-ABS-KEY ( hospital* W/2 home) OR TITLE-ABS-KEY ( "early supported discharge" ) OR TITLE-ABS-KEY (home W/1 care) OR TITLE-ABS-KEY ( "virtual ward" ) OR TITLE-ABS-KEY ( "virtual hospital" ) OR TITLE-ABS-KEY ( "bed substitution" ) ) |
|  | #3 | (TITLE-ABS-KEY ( "recommendation" ) OR TITLE-ABS-KEY ( "polic*" ) OR TITLE-ABS-KEY ( "implement*" ) OR TITLE-ABS-KEY ( "requirement" ) OR TITLE-ABS-KEY ( "guideline" ) ) |
|  | #4 | Combined Search:  ( TITLE-ABS-KEY ( "recommendation" ) OR TITLE-ABS-KEY ( "policy" ) OR TITLE-ABS-KEY ( "implement*" ) OR TITLE-ABS-KEY ( "guideline" ) AND TITLE-ABS-KEY ( "geriatric" ) OR TITLE-ABS-KEY ( "family practice" ) OR TITLE-ABS-KEY ( "internal medicine" ) OR TITLE-ABS-KEY ( "inpatient" ) OR TITLE-ABS-KEY ( "acute disease" ) OR TITLE-ABS-KEY ( "subacute care" ) AND TITLE-ABS-KEY ( hospital* W/2 home ) OR TITLE-ABS-KEY ( "early supported discharge" ) OR TITLE-ABS-KEY ( home W/1 care ) OR TITLE-ABS-KEY ( "virtual ward" ) OR TITLE-ABS-KEY ( "virtual hospital" ) OR TITLE-ABS-KEY ( "bed substitution" ) ) AND PUBYEAR > 2013 AND PUBYEAR < 2024 AND ( LIMIT-TO ( SUBJAREA , "MEDI" ) OR LIMIT-TO ( SUBJAREA , "NURS" ) OR LIMIT-TO ( SUBJAREA , "SOCI" ) OR LIMIT-TO ( SUBJAREA , "HEAL" ) ) AND ( LIMIT-TO ( LANGUAGE , "English" ) )  1,185 documents found |
| Web of Science | #1 | TS=(hospital* NEAR/2 home OR "early supported discharge" OR home NEAR/1 care OR "virtual ward" OR "virtual hospital" OR "bed substitution" ) |
|  | #2 | TS=("geriatric" OR "family medicine" OR "internal medicine" OR "inpatient" OR "acute disease" OR "subacute care" ) |
|  | #3 | TS=("recommendation" OR "polic*" OR "implement*" OR "requirement" OR "guideline" ) |
|  | #4 | DOP=(2014/2023) |
|  | #5 | #1 AND #2 AND #3 AND #4  Results: 508 |
| Internet Search |  | (“Hospital in the home" OR "Hospital at home" OR "home hospital" OR "early supported discharge" OR"virtual ward" OR "virtual hospital" OR "bed substitution" OR "hospital based home care”) AND (recommendation OR policy OR implementation OR guide OR toolkit) AND ("inpatient" OR "acute") |
